# Therapeutical and Nutraceutical Roles of Cyanobacterial Tetrapyrrole Chromophore: Recent Advances and Future Implications

**DOI:** 10.3389/fmicb.2022.932459

**Published:** 2022-07-19

**Authors:** Kshetrimayum Birla Singh, Kaushalendra, Jay Prakash Rajan

**Affiliations:** ^1^Department of Life Science, Manipur University, Imphal, India; ^2^Department of Zoology, Pachhunga University College, Mizoram University, Aizawl, India; ^3^Department of Chemistry, Pachhunga University College, Mizoram University, Aizawl, India

**Keywords:** tetrapyrrole, antioxidants, phycobiliprotein, phycocyanin, therapeutic agent

## Abstract

Cyanobacteria have attracted the attention of researchers because of their promising role as primary and secondary metabolites in functional food and drug design. Due to an ever-increasing awareness of health and the use of natural products to avoid the onset of many chronic and lifestyle metabolic diseases, the global demand for the use of natural drugs and food additives has increased in the last few decades. There are several reports about the highly valuable cyanobacterial products such as carotenoids, vitamins, minerals, polysaccharides, and phycobiliproteins showing antioxidant, anti-cancerous, anti-inflammatory, hypoglycemic, and antimicrobial properties. Recently, it has been shown that allophycocyanin increases longevity and reduces the paralysis effect at least in *Caenorhabditis elegans*. Additionally, other pigments such as phycoerythrin and phycocyanin show antioxidative properties. Because of their high solubility in water and zero side effects, some of the cyanobacterial tetrapyrrole derivatives, i.e., pigments, facilitate an innovative and alternative way for the beverage and food industries in place of synthetic coloring agents at the commercial level. Thus, not only are the tetrapyrrole derivatives essential constituents for the synthesis of most of the basic physiological biomolecules, such as hemoglobin, chlorophyll, and cobalamin, but also have the potential to be used for the synthesis of synthetic compounds used in the pharmaceutical and nutraceutical industries. In the present review, we focused on the different aspects of tetrapyrrole rings in the drug design and food industries and addressed its remaining limitations to be used as natural nutrient supplements and therapeutic agents.

## Introduction

Cyanobacteria are the earliest inhabitants of the earth and flourish in almost every habitat such as soil, rock, fresh water, and marine water, including some of the toughest environments such as hot springs. The secret behind their universal distribution in each and every corner of the globe lies in their ability to produce a wide range of bioactive compounds that evolved to protect them from various exogenous and endogenous environmental insults. Recently, with the advanced biotechnological route, humans have started to use the same cyanobacterial compounds in agriculture, pharmacology, and formulation of functional foods because of their ability to be used as an alternative source of cytoprotective, nutritive, and therapeutic compounds that may be present at suboptimal levels in human body under certain pathological conditions (Fernández-Rojas et al., 2014a; Pagels et al., 2019). This is the reason why researchers have started to pay attention to extracting the novel bioactive components from cyanobacteria at a low cost and in an efficient way. It has been established already that most cyanobacterial compounds have tetrapyrrole rings in the heart of their supermolecular structure. Tetrapyrrole pigments are closely related to bilirubin molecules showing potent antioxidative and anti-proliferative properties when used as food supplements (Dillon et al., 1995; Konícková et al., 2014). There are several reports that most of the cyanobacterial species are laden with a higher quantity of essential amino acids, proteins, vitamins, flavonoids, unsaturated fatty acids, vitamins, and minerals as compared to traditional food such as milk, vegetables, fruits, soybean, egg, fish, and meat (Pervushkin et al., 2001; Khan et al., 2005; Jin et al., 2021). Therefore, due to their enriched nutritional value, the extraction of bioactive components from cyanobacteria has been proven as a boon for health. They serve as a sustainable source of raw material for drug development for the cure of various diseases and nutrient-related deficiencies (Ferris and Hirsch, 1991; Pyne et al., 2001; Soares et al., 2015). In recent years, researchers are focused on the biocompatibility of these compounds, especially with proteins in the nanosystem of host cells, to maintain human’s cellular health along with the correction of lifestyle and dietary-related diseases.

One of the most important natural antioxidant compounds present in cyanobacteria is its pigment, a tetrapyrrole linear derivative, along with a rich source of vitamins and minerals; however, their primary biological role is to do photosynthesis, thus contributing to the production of a major amount of biomass in the ecosystem (Romay et al., 1998, 2003; Romay and Gonzalez, 2000; Remirez et al., 2002; Eriksen, 2008; Thangam et al., 2013; Zhang et al., 2022). Among the functional components identified in cyanobacteria, natural pigments such as chlorophylls, carotenoids, and phycobilins have received attention due to their linear tetrapyrrolic structure. The tetrapyrrolic derivatives have many potential applications in the cosmetics, pharmaceutical food, and textile industries. In fact, the derivatives of tetrapyrrole form a battery of novel bioactive compounds that may be used to improve the human health as they show the antibacterial, antiviral, antiarthritic, cytotoxic, and immunoregulative properties (Ou et al., 2010; Zheng et al., 2013; Young et al., 2016). The aim of the present review was to discuss the use of tetrapyrrole pigment of cyanobacterial origin for nutraceutical and therapeutical purposes.

## Tetrapyrrole Pigment in the Nutraceutical Industry

Due to the ever-increasing population throughout the entire globe, it has become a challenging task to help the population maintain a balanced nutritional state. Since agricultural production cannot be increased with the pace of the increasing population, researchers now have started to look toward alternative food sources to fulfill the global demand for healthier food products. They have started to focus on the cyanobacteria being a rich source of proteins, the most important food component for a balanced diet. One of the cyanobacterial species, i.e., *Spirulina*, has made its way to the dining table because of its high nutritional values. The other bioactive compounds present in *Spirulina* show antioxidative, antimicrobial, anti-cancer, antiviral, and anti-inflammatory activities (Hayashi et al., 1994; Bath and Madyastha, 2000; Carmichael et al., 2000; Hirata et al., 2000). There is scientific evidence that *Spirulina*’s is a novel food supplement and is well-recognized for preventing and managing certain diseases such as hypercholesterolemia and cancer. A number of cyanobacterial species host a wide range of secondary metabolites and natural pigments such as β carotene, scytonemin, phycoerythrin, phycocyanin, and phycobilisomes. These pigments have been proved as healthy bioactive constituents of cyanobacterial species and are being utilized by researchers’ disease diagnostics, supplementary food, and herbal medicines (Sielaff et al., 2006; Tan, 2007; Gademann and Portmann, 2008; Ghosh et al., 2016). Phycocyanin extracted from *S. platensis* widely used as a cosmetic colorant and food additive. Recently, researchers have moved their focus to the use of cyanobacterial pigments such as phycobiliprotein as food supplements in the nutraceutical industry. The dried contents of *Arthrospira platensis* may play an important role in functional foods because of the bioactive compounds showing immunoregulative and antioxidative properties. *A. platensis* also suppresses the inflammation, viral infection, cancer progression, and maturity of cholesterol-related diseases (Jensen et al., 2001; Matsui et al., 2012).

## Antitumor Effect of Tetrapyrrole Pigment

Cancer is the worst result of harmful mutations in the nuclear and mitochondrial genes along with the foul games of free radicals in the cellular ecosystem, causing several unwelcomed molecular changes. Many of the cyanobacterial compounds are reported to check cancer and tumor progression. Natural compounds obtained from *Spirulina platensis* exhibit antiproliferative properties (Mysliwa-Kurdziel and Solymosi, 2017). The possible paths of this mechanism lie in the activity of tetrapyrrole compounds, such as phycobiliproteins and phycocyanin, being nucleophilic in nature and thus being able to prevent the cellular damage by neutralizing the excess reactive oxygen species. Thangam et al. (2013) investigated in detail the effectiveness of phycobiliprotein in the inhibition of colon cancer (HT-29) and lung cancer (A549). Gupta and Gupta (2012) suggested that phycobiliprotein may have suppressive effects on TPA-induced tumors on mice skin. Jiang et al. (2017) reported that phycocyanin is effective in checking the entry of HeLa cancerous cells into the G2 phase of the cell cycle. Recently, Liu et al. (2012) noted that phycocyanin promotes the release of cytochrome c (Cyt c), which in turn repair the damaged DNA and promote apoptosis in Hepal-6 cells. A cyanobacterial tetrapyrrole pigment also ameliorates the cellular damage in normal cells done by chemotherapy and radiotherapy by reducing the oxidative stress and enhancing the antioxidative defense system.

## Nephroprotective Effect of Tetrapyrrole Pigment

Kidney disease and failure occur under several pathophysiological conditions such as severe diabetes, pesticides, and heavy metal contamination (nephrotoxins) and due to sepsis syndrome, cardiorenal syndrome, and obstruction of the urinary tract. Renal diseases are diagnosed by the Renal Function Test in which abnormal serum creatinine level and urine output are the most important parameters. It is reported that oxidative load in such critical conditions acts synergistically enhancing nephrotoxicity. The nephroprotective effects of phycoerythrins and phycobiliproteins, which contain tetrapyrrole, play a key role in lowering oxidative stress and minimizing damage to renal cells. *Spirulina* and its extracted components have the potential to improve the glomerular functions in renal cells by maintaining the normal redox environment under oxidative stress conditions. Natural pigments from cyanobacteria are involved in the regulation of mitochondrial activity especially in renal cells *via* energy production and control the apoptosis progression. Experimental pieces of evidence suggest that oxidative load is one of the major causes of nephrotoxicity and renal dysfunction which can be determined by measurement of biomarkers by the level of malondialdehyde, glutathione, and activity of superoxide dismutase, and catalase. It is quite evident that *Spirulina* extract can diminish the level of malondialdehyde and glutathione while enhancing the biosynthesis and activity of superoxide dismutase and catalase in mesangial renal cells restoring the normal functions of the kidney. Phycocyanins from *Spirulina* are reported to modify the physicochemical forces such as oncotic and hydrostatic pressures which are suspected to influence the pressure along with the glomerular filter (Europa et al., 2010; Lim et al., 2012; Fernández-Rojas et al., 2014b; Liu et al., 2017; Mysliwa-Kurdziel and Solymosi, 2017). Phycocyanins from *Spirulina* also prevent the formation of oxalic acid which may form the calcium oxalate in renal calculi. Additionally, phycocyanins down-regulate the ROS production and lipid peroxidation in kidney cells (Riss et al., 2007; Zheng et al., 2013; Farooq et al., 2014).

## Hepatoprotective Effect of Tetrapyrrole Pigment

Liver diseases are caused by different etiological conditions such as viral infections, metabolic syndrome, alcohol and drug abuse, autoimmune diseases, and toxins, including pesticides, heavy metal contamination, and microplastics. The cyanobacterial extract is reported to have excellent hepatoprotective bioactive compounds that inhibit the kinases activity, a key protein of the cell cycle that cures the hyperproliferative disease without any chemical toxicity. Vadiraja et al. (1998) noted the pharmacological activities of phycocyanin and reported that it could neutralize the effect of ROS in liver cells. Cyanobacterial phycocyanin is also branded to control the synthesis and activity of many hepatic enzymes such as microsomal cytochrome P450, aminopyrine-N-demethylase, and glucose-6-phosphatase. Phycocyanin can suppress inflammation by blocking the hepatocyte growth factor (Vadiraja et al., 1998; Riss et al., 2007). Further, along with phycocyanin, phycocyanobilin also exhibits free radical scavenging activity (Bath and Madyastha, 2000; Ou et al., 2010).

## Cardio-Protective Effect of Tetrapyrrole Pigment

Arterial thrombotic stress such as myocardial infarction and heart stroke is the foremost cause of cardiovascular diseases, ranking at the first position in the list of causes of human mortality worldwide. The platelet activity, particularly the aggregation and activation of platelets, significantly controls the debut of atherothrombotic disease. In traditional and modern medical practices, cyanobacterial extracts, particularly from *Spirulina*, are known to lower the serum cholesterol level as phycocyanin is involved in regulating the cholesterol absorption and the bile acid reabsorption process (Iwata et al., 1990; Shibata et al., 2001; Nagaoka et al., 2005; Radibratovic et al., 2016). Also, phycocyanin’s constituent compounds, a class of covalently bonded open-chain tetrapyrrole derivatives, work as a pro-drug after its break down due to gastrointestinal enzymes and extend its therapeutic effects (Radibratovic et al., 2016). Tetrapyrrole pigments are well-established in preventing redox environmental disturbances, thus increasing the myocardial enzymatic activity. On the one hand, phycocyanin downregulates the signaling pathways, leading to inflammation by inhibiting the phospho-NFκB p65 enzyme while decreasing the synthesis of mRNAs of proinflammatory cytokines (Hao et al., 2018). On the other hand, the same pigment is also known to promote the heme oxygenase-1 molecular pathway enhancing the anti-inflammatory processes. It also diminishes the activity of caspase, thus minimizing the cell death but endorsing the synthesis of antioxidant enzymes (Gao et al., 2016; Kim et al., 2018).

## Antidiabetic Effect of Tetrapyrrole Pigment

Diabetes mellitus is a metabolic disorder in which lifelong hyperglycemia is manifested due to a decline in glycogen synthesis, leading to abnormal glucose levels in the blood. Prolonged hyperglycemia leads to other chronic diseases such as heart disease, kidney failure, and blindness. In 2010, Ou et al. showed that cyanobacterial pigments, especially phycocyanin, significantly enhance the muscular and hepatic glycogen synthesis, restoring the glucose homeostasis. Insulin secretion is directly related to the bio efficiency of α cells whose hormonal synthesis and secretion decrease significantly under stress, hypertension, inflammation, obesity, and aging. deKoning et al. (2008) reported that phycocyanin can control the size of the endocrine portion of the pancreas, influencing insulin secretion significantly (Yu et al., 2013). Ghosh et al. (2016) found that glucose metabolism is influenced by oxidative stress, which in turn is dropped off by the free radical scavenging activity of cyanobacterial pigments. Further, extracted pigments from *Synechocystis*, *Lyngbya*, and *Microcoleus* species inhibit the α-glycosidase and α-amylase activities, leading to hypoglycemic conditions. Additionally, the α-amylase and α-glucosidase inhibitions also decline the occurrence of diarrhea and flatulence, both of which are linked to indigestion of food in the gastrointestine tract. Cyanobacterial pigments enhance the digestion *via* fermentation done by lactic acid bacteria. The crude as well as purified phycoerythrin and phycocyanin play a dual role as hypoglycemic and antioxidative activities and could be used as food additives in food industries.

## Conclusion

The successful use of cyanobacteria in nutraceutical and pharmaceutical applications will depend on its novel tetrapyrrole-derived bioactive compounds which encompass antiallergic, anticarcinogenic, antibacterial, anticoagulant, antifungal, antihypertensive, anti-inflammatory, antinociceptive, antioxidant, antipyretic, cholesterol-lowering, hepatoprotective, and immune enhancement properties. Positive health effects have been related to the fact that the structure of phycocyanorubin and bilirubin resembles each other. In addition to their valuable role in medicinal and functional food, this group of natural pigments from cyanobacteria represents an attractive source of bioactive sustainable compounds. Tetrapyrrole-derived compounds in cyanobacteria have been used as a food additive in chewing gum, candies, food supplements, beverages such as soft drinks, and cosmetics products such as lipsticks and eyeliners. They also show therapeutic properties such as antitumor, nephroprotective, hepatoprotective, anti-diabetes, and antioxidant activities. These scientific studies announce that the consumption of edible cyanobacteria may be a safe alternative approach in the therapeutic and nutraceutical industries without any side effect.

## Author Contributions

JR wrote this article. Ka collected the research article. KS edited and corrected this article. All authors contributed to the article and approved the submitted version.

## Conflict of Interest

The authors declare that the research was conducted in the absence of any commercial or financial relationships that could be construed as a potential conflict of interest.

## Publisher’s Note

All claims expressed in this article are solely those of the authors and do not necessarily represent those of their affiliated organizations, or those of the publisher, the editors and the reviewers. Any product that may be evaluated in this article, or claim that may be made by its manufacturer, is not guaranteed or endorsed by the publisher.

## References

[B1] Bath V. B. Madyastha K. M. (2000). C-phycocyanin: a potent peroxyl radical scavenger *in vivo* and *in vitro*. *Biochem. Biophys. Res. Commun.* 275 20–25. 10.1006/bbrc.2000.3270 10944434

[B2] Carmichael W. W. Drapeau C. Anderson D. M. (2000). Harvesting of *Aphanizomenon* flos-aquae Ralfs ex Born & Flah. var. flos-aquae (*Cyanobacteria*) from Klamath Lake for human dietary use. *J. Appl. Phycol.* 12 585–595. 10.1023/A:1026506713560

[B3] deKoning E. J. Bonner-Weir S. Rabelink T. J. (2008). Preservation of beta-cell functions by targeting beta-cell mass. *Trends Pharmacol. Sci.* 29 218–227. 10.1016/j.tips.2008.02.001 18359095

[B4] Dillon J. C. Phuc A. P. Dubacq J. P. (1995). Nutritional value of the alga Spirulina. *World Rev. Nutr. Diet.* 77 32–46. 10.1159/000424464 7732699

[B5] Eriksen N. T. (2008). Production of phycocyanin–a pigment with applications in biology, biotechnology, foods and medicine. *Appl. Microbiol. Biotechnol.* 80 1–14. 10.1007/s00253-008-1542-y 18563408

[B6] Europa C. E. Butron R. O. Gallardo-Casas C. A. Blas-Valdivia V. Pineda-Reynoso M. Olvera-Ramírez R. (2010). Phycobiliproteins from Pseudanabaena tenuisrich in c-phycoerythrin protect against HgCl2-caused oxidative stress and cellular damage in the kidney. *J. Appl. Phycol.* 22 495–501. 10.1007/s10811-009-9484-z

[B7] Farooq S. M. Boppana N. B. Devarajan A. Sekaran S. D. Shankar E. M. (2014). C-phycocyanin confers protection against oxalate-mediated oxidative stress and mitochondrial dysfunctions in MDCK cells. *PLoS One* 9:e93056. 10.1371/journal.pone.0093056 24691130 PMC3972226

[B8] Fernández-Rojas B. Medina-Campos O. N. Hernández-Pando R. Negrette-Guzmán M. Huerta-Yepez S. Pedraza-Chaverri J. (2014a). C-Phycocyanin prevents cisplatin-induced nephro-toxicity through inhibition of oxidative stress. *Food Funct.* 5 480–490. 10.1039/c3fo60501a 24503583

[B9] Fernández-Rojas B. Hernández-Juárez J. Pedraza-Chaverri J. (2014b). Nutraceutical properties of Phycocyanin. *J. Funct. Foods* 11 375–392. 10.1016/j.jff.2014.10.011

[B10] Ferris M. J. Hirsch C. F. (1991). Method for isolation and purification of *Cyanobacteria*. *Appl. Environ. Microbiol.* 57 1448–1452. 10.1128/aem.57.5 16348486 PMC182968

[B11] Gademann K. Portmann C. (2008). Secondary metabolites from *Cyanobacteria*: complex structure and powerful bioactivities. *Curr. Org. Chem.* 12 326–330. 10.2174/138527208783743750

[B12] Gao Y. Liu C. Wan G. Wang X. Cheng X. Ou Y. (2016). Phycocyanin prevents methylglyoxal-induced mitochondrial-dependent apoptosis in INS-1 cells by Nrf2. *Food Funct.* 7 1129–1137. 10.1039/c5fo01548k 26805012

[B13] Ghosh T. Bhayani K. Paliwal C. Maurya R. Chokshi K. Pancha I. (2016). *Cyanobacterial* pigments as natural anti-hyperglycemic agents: an *in vitro* study. *Front. Marine Sci.* 3 146. 10.3389/fmars.2016.00146

[B14] Gupta N. K. Gupta K. P. (2012). Effects of C-Phycocyanin on the representative genes of tumor development in mouse skin exposed to 12-O-tetradecanoyl-phorbol-13-acetate. *Environ. Toxicol. Pharmacol.* 34 941–948. 10.1016/j.etap.2012.08.001 22986104

[B15] Hao S. Yan Y. Huang W. Gai F. Wang J. Liu L. (2018). C-Phycocyanin reduces inflammation by inhibiting NF-κB activity through down regulating PDCD5 in lipopolysaccharide-induced RAW 264.7 macrophages. *J. Funct. Foods* 42 21–29.

[B16] Hayashi O. Katoh T. Okuwaki Y. (1994). Enhancement of anti-body production in mice by dietary *Spirulina platensis*. *J. Nutr. Sci. Vitaminol.* 40 431–441. 10.3177/jnsv.40.431 7891204

[B17] Hirata T. Tanaka M. Ooike M. Tsunomura T. Sakaguchi M. (2000). Antioxidant activities of phycocyanobilin prepared from *Spirulina platensis*. *J. Appl. Phycol.* 12 435–439.

[B18] Iwata K. Inayama T. Kato T. (1990). Effects of *Spirulina platensis* on plasma lipoprotein lipase activity in fructose-induced hyperlipidemic rats. *J. Nutr. Sci. Vitaminol.* 36 165–171. 10.3177/jnsv.36.165 2117648

[B19] Jensen G. S. Ginsberg D. I. Drapeau C. (2001). Blue-green algae as an immuno-enhancer and biomodulator. *J. Am. Nutraceut. Ass.* 3 24–30. 10.3389/fenvs.2018.00007

[B20] Jiang L. Wang Y. Yin Q. Liu G. Liu H. Huang Y. (2017). Phycocyanin: a Potential Drug for Cancer Treatment. *J. Cancer* 8 3416–3429. 10.7150/jca.21058 29151925 PMC5687155

[B21] Jin H. Wang Y. Zhao P. Wang L. Zhang S. Meng D. (2021). Potential of producing flavonoids using *Cyanobacteria* as a sustainable chassis. *J. Agric. Food Chem.* 69 12385–12401. 10.1021/acs.jafc.1c04632 34649432

[B22] Khan Z. Bhadouria P. Bisen P. (2005). Nutritional and therapeutic potential of spirulina, Curr. Pharm. *Biotechnology* 6 373–379. 10.2174/138920105774370607 16248810

[B23] Kim K. M. Lee J. Y. Im A.-R. Chae S. (2018). Phycocyanin protects against UVB-induced apoptosis through the PKC α/βII-Nrf-2/HO-1 dependent pathway in human primary skin cells. *Molecules* 23:478. 10.3390/molecules23020478 29470442 PMC6017183

[B24] Konícková R. Vanková K. Vaníková J. Vánová J. Muchová L. Subhanová I. (2014). Anti-cancer effects of blue-green alga *Spirulina platensis*, a natural source of bilirubin-like tetrapyrrolic compounds, Annals of Hept. *Ann. Hepatol.* 13 273–283. 24552870

[B25] Lim B. J. Jeong J. Y. Chang Y. K. Na K. R. Lee K. W. Shin Y. T. (2012). C-phycocyanin attenuates cisplatin-induced nephrotoxicity in mice. *Ren. Fail.* 34 892–900. 10.3109/0886022X.2012.690925 22681485

[B26] Liu C. Fu Y. Li C. E. Chen T. Li X. (2017). Phycocyanin-functionalized selenium nanoparticles reverse palmitic acid-induced pancreatic b-cell apoptosis by enhancing cellular uptake and blocking reactive oxygen species (ROS)-mediated mitochondria dysfunction. *J. Agri. Food Chem.* 65 4405–4413. 10.1021/acs.jafc.7b00896 28510423

[B27] Liu S. F. Wang T. Y. Gao Y. (2012). Effects of phycocyanin on Hepa1-6 cells and immune function of mice bearing tumor. *Chin. J. Public Health* 3 342–349.

[B28] Matsui K. Nazifi E. Hirai Y. Wada N. Matsugo S. Sakamoto T. (2012). The *Cyanobacterial* UV-absorbing pigment scytonemin displays radical-scavenging activity. *J. Gen. Appl. Microbiol.* 58 137–144. 10.2323/jgam.58.137 22688245

[B29] Mysliwa-Kurdziel B. Solymosi K. (2017). Phycobilins and phycobiliproteins used in food industry and medicine. *Mini. Rev. Med. Chem.* 17 1173–1193. 10.2174/1389557516666160912180155 27633748

[B30] Nagaoka S. Shimizu K. Kaneko H. Shibayama F. Morikawa K. Kanamura Y. (2005). Novel protein C-phycocyanin plays a crucial role in the hypocholesterolomic action of *Spirulina platenis* concentrate in rats. *J. Nutr.* 135 2425–2430. 10.1093/jn/135.10.2425 16177207

[B31] Ou Y. Zheng S. Lin L. Jiang Q. Yang X. (2010). Protective effect of C-phycocyanin against carbon tetrachloride-induced hepatocyte damage *in vitro* and *in vivo*. *Chem. Bio. Interact.* 185 94–100. 10.1016/j.cbi.2010.03.013 20227401

[B32] Pagels F. Guedes A. C. Amaro H. M. Kijjoa A. Vasconcelos V. (2019). Phycobiliproteins from *Cyanobacteria*: chemistry and Biotechnological Applications. *Biotechnol. Adv.* 37 422–443. 10.1016/j.biotechadv.2019.02.010 30797095

[B33] Pervushkin S. V. Voronin A. V. Kurkin V. A. Sokhina A. A. Shatalaev I. F. (2001). Proteins from *Spirulina platensis* biomass. *Chem. Nat. Comp.* 37 476–480.

[B34] Pyne P. K. Bhattacharjee Srivastav P. (2001). Microalgae (*Spirulina platensis*) and its bioactive molecules: review. *Indian J. Nutr.* 4 160–165.

[B35] Radibratovic M. Minic S. Stanic-Vucinic D. Nikolic M. Milcic M. Cirkovic Velickovic T. (2016). Stabilization of human serum albumin by the binding of phycocyanobilin, a bioactive chromophore of blue-green alga Spirulina: molecular dynamics and experimental study. *PLoS One* 11:e0167973. 10.1371/journal.pone.0167973 27959940 PMC5154526

[B36] Remirez D. Ledón N. González R. (2002). Role of histamine in the inhibitory effects of phycocyanin in experimental models of allergic inflammatory response. *Mediators Inflamm.* 11 81–85. 10.1080/09629350220131926 12061428 PMC1781653

[B37] Riss J. Décordé K. Sutra T. Delage M. Baccou J. C. Jouy N. (2007). Phycobiliprotein C-phycocyanin from *Spirulina platensisis* powerfully responsible for reducing oxidative stress and NADPH oxidase expression induced by an atherogenic diet in hamsters. *J. Agri. Food Chem.* 55 7962–7967. 10.1021/jf070529g 17696484

[B38] Romay C. Armesto J. Remirez D. Gonzalez R. Ledon L. Garcia I. (1998). Antioxidant and anti-inflammatory properties of C-phycocyanin from blue-green algae. *Inflamm. Res.* 47 36–41. 10.1007/s000110050256 9495584

[B39] Romay C. Gonzalez R. (2000). Phycocyanin is an antioxidant protector of human erythrocytes against lysis by peroxyl radicals. *J. Pharm. Pharmacol.* 52 367–368. 10.1211/0022357001774093 10813544

[B40] Romay C. H. González R. Ledón N. Remirez D. Rimbau V. (2003). C-phycocyanin: a biliprotein with antioxidant, anti-inflammatory and neuroprotective effects. *Curr. Protein Pept. Sci.* 4 207–216. 10.2174/1389203033487216 12769719

[B41] Shibata S. Oda K. Onodera-Masuoka N. Matsubara S. Kikuchi Hayakawa H. Ishikawa F. (2001). Hypocholesterolemic effect of indigestible fraction of Chlorella regularis in cholesterol-fed rats. *J. Nutr. Sci. Vitaminol.* 47 373–377. 10.3177/jnsv.47.373 11922110

[B42] Sielaff H. Christiansen G. Schwecke T. (2006). Natural products from *Cyano-bacteria*: exploiting a new source for drug discovery. *IDrugs* 9 119–127. 16523402

[B43] Soares A. R. Engene N. Gunasekera S. P. Sneed J. M. Paul V. J. (2015). Carriebowlinol, an antimicrobial tetrahydroquinolinol from an assemblage of marine *Cyanobacteria* containing a novel taxon. *J. Nat. Prod.* 78 534—538. 10.1021/np500598x 25536090

[B44] Tan L. T. (2007). Bioactive natural products from marine *Cyanobacteria* for drug discovery. *Phytochemistry* 68 954–979. 10.1016/j.phytochem.2007.01.012 17336349

[B45] Thangam R. Suresh V. Asenath P. W. Rajkumar M. Senthilkumar N. Gunasekaran P. (2013). C-Phycocyanin from Oscillatoria tenuis exhibited an antioxidant and *in vitro* antiproliferative activity through induction of apoptosis and G0/G1 cell cycle arrest. *Food Chem.* 140 262–272. 10.1016/j.foodchem.2013.02.060 23578642

[B46] Vadiraja B. B. Gaikwad N. W. Madyastha K. M. (1998). Hepatoprotective effect of C-phycocyanin: protection for carbon tetrachloride and R-(+)-pulegone-mediated hepatotoxicty in rats. *Bioch. Biophy. Res. Commun.* 249 428–431. 10.1006/bbrc.1998.9149 9712713

[B47] Young I. Chuang S. Hsu C. Sun Y. Lin F. (2016). C-phycocyanin alleviates osteoarthritic injury in chondrocytes stimulated with H2O2 and compressive stress. *Int. J. Biol. Macromol.* 93 852–859. 10.1016/j.ijbiomac.2016.09.051 27642127

[B48] Yu O. Lin L. Xuegan Y. Qin P. Xiaodong C. (2013). Antidiabetic potential of phycocyanin: effects on KKAy mice. *Pharmac. Biol.* 51 539–544. 10.3109/13880209.2012.747545 23368938

[B49] Zhang L. Kong D. Huang J. Wang Q. Shao L. (2022). The therapeutic effect and the possible mechanism of C-phycocyanin in lipopolysaccharide and seawater-induced acute lung injury. *Drug Des. Devel. Ther.* 6 1025–1040. 10.2147/DDDT.S347772 35418745 PMC8995161

[B50] Zheng J. Inoguchi T. Sasaki S. Maeda Y. McCarty M. F. Fujii M. (2013). Phycocyanin and phycocyanobilin from *Spirulina platensis* protect against diabetic nephropathy by inhibiting oxidative stress. *Am. J. Physiol. Regul. Integr. Comp. Physiol.* 304 110–120. 10.1152/ajpregu.00648.2011 23115122

